# Are there effects of light exposure on daytime sleep for rotating shift nurses after night shift?: an EEG power analysis

**DOI:** 10.3389/fnins.2024.1306070

**Published:** 2024-03-27

**Authors:** Soonhyun Yook, Su Jung Choi, Cong Zang, Eun Yeon Joo, Hosung Kim

**Affiliations:** ^1^USC Stevens Neuroimaging and Informatics Institute, Keck School of Medicine of USC, University of Southern California, Los Angeles, CA, United States; ^2^Graduate School of Clinical Nursing Science, Sungkyunkwan University, Seoul, Republic of Korea; ^3^Department of Neurology, Neuroscience Center, Samsung Medical Center, Samsung Biomedical Research Institute, School of Medicine, Sungkyunkwan University, Seoul, Republic of Korea

**Keywords:** shift work, sleep EEG, light exposure, electrophysiology, EEG spectral analysis, circadian misalignment

## Abstract

**Introduction:**

Night-shift workers often face various health issues stemming from circadian rhythm shift and the consequent poor sleep quality. We aimed to study nurses working night shifts, evaluate the electroencephalogram (EEG) pattern of daytime sleep, and explore possible pattern changes due to ambient light exposure (30 lux) compared to dim conditions (<5 lux) during daytime sleep.

**Moethods:**

The study involved 31 participants who worked night shifts and 24 healthy adults who had never worked night shifts. The sleep macro and microstructures were analyzed, and electrophysiological activity was compared (1) between nighttime sleep and daytime sleep with dim light and (2) between daytime sleep with dim and 30 lux light conditions.

**Results:**

The daytime sleep group showed lower slow or delta wave power during non-rapid eye movement (NREM) sleep than the nighttime sleep group. During daytime sleep, lower sigma wave power in N2 sleep was observed under light exposure compared to no light exposure. Moreover, during daytime sleep, lower slow wave power in N3 sleep in the last cycle was observed under light exposure compared to no light exposure.

**Discussion:**

Our study demonstrated that night shift work and subsequent circadian misalignment strongly affect sleep quality and decrease slow and delta wave activities in NREM sleep. We also observed that light exposure during daytime sleep could additionally decrease N2 sleep spindle activity and N3 waves in the last sleep cycle.

## 1 Introduction

Night-shift workers, defined as individuals who generally work between 21:00 and 08:00 (Wright et al., 2013), comprise 3.3% of the total working population (May, 2011). They often face various health issues stemming from poor sleep quality, which is affected by reduced time in bed, outside noise, and family interruptions (May, 2011). The reduced sleep quality associated with night-shift work leads to excessive sleepiness and impaired alertness and may negatively impact patient care and safety (Kecklund and Axelsson, 2016; Alfonsi et al., 2021).

Circadian rhythms, primarily concurring with the 24-h light/dark cycle, are regulated by the suprachiasmatic nuclei (SCN) located in the hypothalamus (Wright et al., 2013; Alshahrani et al., 2017). This system typically encourages wakefulness during the day and sleep at night (Wright et al., 2013; Alshahrani et al., 2017). However, owing to circadian rhythm misalignment, night-shift workers may face difficulties in maintaining quality daytime sleep. In particular, exposure to strong lights during nighttime work has been associated with a disrupted circadian system (Yoon et al., 2002; Knufinke et al., 2021), lowered sleep efficiency (Martin et al., 2015), decreased time in bed (Choi and Joo, 2016), and attenuated alertness (or wakefulness) after waking (Yoon et al., 2002; Chang et al., 2015; Esaki et al., 2019). Furthermore, light exposure during sleep can impair glucose homeostasis, potentially via increased sympathetic nervous system activation (Mason et al., 2022). Thus, two questions arise: (1) *Whether daytime sleep for night shift workers is different from nighttime sleep for daytime workers? (2) If this is true, is the difference due to circadian rhythm shift or light exposure during sleep or both?*

The effects of circadian rhythm shifts on daytime sleep in night-shift workers have been investigated using objective sleep parameters based on polysomnography (PSG) and subjective measures using self-report questionnaires or sleep diary. These studies showed that the main clinical characteristics of people with sleep disturbance from night-shift work are shorter sleep duration and sleepiness (Wright et al., 2013). In the analysis of the effect of light exposure during daytime sleep, PSG did not reveal significant changes in those working 12-h night shifts (Choi et al., 2019). This suggests that analysis of the standard PSG parameters may not fully explain the effects of ambient light on daytime sleep. Therefore, more sensitive quantitative markers are required to elucidate the effects of light exposure.

In sleep electroencephalography (EEG), several types of characteristic field potential oscillations act as important biomarkers for assessing physiological brain activity in relation to different sleep stages. Slow wave sleep (SWS), with a peak frequency of ~0.8 Hz in humans, occurs primarily in non-rapid eye movement (NREM) N3 sleep (Carlson, 2013; Gage and Baars, 2018). Sleep spindles, brief and powerful bursts originating from specific oscillations generated in thalamic circuits, occur mainly in N2 sleep, with a synchronous 12–15 Hz neuronal firing rate (Manoach and Shinn, 2013; Siclari and Tononi, 2016). Such EEG features may further characterize brain activities that alter during sleep exposed to ambient light. For example, nighttime sleep with lights on was associated with decreased theta power during rapid eye movement (REM) sleep and reductions in slow, delta, and spindle power during NREM sleep (Cho et al., 2013). This suggests that light exposure may lead to less deep nighttime sleep. However, it remains unclear whether light exposure adversely affects daytime sleep in night shift workers. It is hypothesized that the effects of light exposure may be different and more complicated during daytime sleep because of the known adverse effects of circadian rhythm shifts on sleep (James et al., 2020; Mentink et al., 2020).

Thus, we aimed to study the pattern of relative power changes of EEG in each cycle and stage of daytime sleep in nurses, after the first night shift in their weekly work schedule, and explore possible pattern changes due to ambient light exposure (30 lux) compared to dim conditions (< 5 lux). We further investigated the temporal dynamics of the relative power of sleep EEG in various brain regions.

## 2 Methods

### 2.1 Participants and study screening

Participants were recruited from two cohorts at the Samsung Medical Center (SMC) in Seoul, South Korea: (a) night shift nurses with daytime sleep and (b) day workers with regular nighttime sleep. We advertised in the hospital cafeteria to recruit participants. All participants were female Koreans and were older than 24 years of age. In case of night shift nurse, they worked in rotating shifts work for more than 1 year at a metropolitan hospital (≥2,000 beds) in Seoul, Republic of Korea. Participants were excluded if they were currently using hypnotics or central nervous system stimulants, had a history of any psychiatric illness or major systemic disease, smoked cigarettes, drank more than 2 glasses per day, or were pregnant or lactating during the study period. Participants who experienced sleep disorders, other than insomnia, such as obstructive sleep apnea with Apnea-hypopnea index equal to or greater than 5 events per hour in PSG, narcolepsy, rapid eye movement sleep behavior disorder, restless legs syndrome, or periodic limb movement disorder confirmed by PSG with a total periodic limb movement index of 15 events per hour or higher and a movement arousal index equal to or greater than 5 events per hour in PSG were also excluded. All participants completed questionnaires on sleep, including the Epworth Sleepiness Scale (ESS) (Johns, 1991), and Insomnia Severity Index (ISI) at their first laboratory visit (Bastien et al., 2001). All participants voluntarily enrolled in this study, and provided written informed consent. All procedures were approved by the Institutional Review Board of Samsung Medical Center (IRB No:2018–05-120 for daytime sleep in shift workers and 2018–10-037 for nighttime sleep in healthy adults) and conducted in accordance with the Declaration of Helsinki.

#### 2.1.1 Night shift nurses with daytime sleep

Thirty-three healthy female nurses who worked two 12-h night shifts were enrolled in this study. Eligible participants had rotating shift work careers, including night shifts, for at least 1 year, and worked a minimum of 24 h per week. The nurses had a two-shift work schedule of D12-D12-N12-N12, followed by four or five rest days (D12 shift from 7:00 a.m. to 7:30 p.m. and N12 shift from 7:00 p.m. to 7:30 a.m., including a 30-min mealtime in each shift). Data collection was conducted between 22 July 2018 and 25 January 2019. A total of 33 shift nurses participated, two of whom participants were excluded due to unmet work schedule requirements. In total, 31 nurses (mean 26.7 ± 3.06 years) were included in the study (Supplementary Figure S1).

#### 2.1.2 Day workers with regular nighttime sleep

Twenty-four healthy female adults (mean 26.9 ± 3.55 years) were identified as the intermediate chronotype as defined by a Morningness-eveningness questionnaire (MEQ) (Horne and Ostberg, 1976) and 7-days sleep diary. The participants slept between 11:00 p.m. and 8:00 a.m. This cohort was recruited and analyzed in our previous study (Park et al., 2020; Jo et al., 2021). Data collection was conducted between 04 December 2018 and 24 August 2019.

#### 2.1.3 Design

For daytime sleep in the night shift nurse group, a one-sample crossover design was used and included 2-day PSG studies: one PSG study was performed under dim light environmental exposure (<5 lux), and another was performed under 30 lux light exposure (at the level of the participants’ eyes) using a bright-controllable light-emitting diode (LED) lamp on the ceiling. Light was maintained throughout the session. The order of light exposure during PSG was determined by 6-block randomization using the R-program. All experiments were performed in a sleep laboratory.

- Sleep with light exposure condition: EEG from PSG during daytime sleep with the light on (30 lux).
- Sleep under dim light conditions: EEG from PSG acquired from the same subjects as above but during another instance of daytime sleep with dim light (<5 lux).

To compare the daytime sleep group described above with the nighttime sleep group, we used our previously published nighttime sleep cohort (described in Section 2.1.2) that was studied in the same laboratory settings (Park et al., 2020; Jo et al., 2021).

#### 2.1.4 Procedures

For daytime sleep, night shift nurses visited the sleep laboratory after completing their first night shift and eating breakfast. Sleep PSG was performed using the Embla N7000 system (Embla, Reykjavik, Iceland). Six-channel EEG signals (F3/F4/C3/C4/O1/O2) were collected with an electrooculogram (EOG), electromyogram, and electrocardiogram to assess sleep stages, recorded at a 200 Hz sampling rate. The usual bedtime of the participants was relatively constant from 9:00 a.m. to 10:00 a.m. and habitual wake-up time varied from 12:00 p.m. to 5:30 p.m. The lights off and start of the sleep study were adjusted to the habitual sleep hours taken after the 12 h night shift. The PSG study was considered completed when the participants woke up of their own will. If the participants failed to wake up at 5:00 p.m., they were woken at 5:30 p.m. for the next night shift and completed the PSG study. Then, the participants were requested to fill out their estimated sleep (subjective total sleep time [TST] and sleep latency). The 2nd PSG study was conducted for each participant during the next shift cycle at the same time. Sleep architecture was scored in 30-s epochs according to the standard criteria by an experienced technician who was blinded to the light conditions.

At night, participants were admitted to the sleep laboratory at approximately 4:00 p.m. They consumed the provided meal and then applied the PSG electrodes. The participants sat on a chair and remained awake with 3,000 or 4,000 K LED light exposure from 6:00 p.m. to 12:00 a.m. The participants went to bed with their usual sleeping arrangements and woke up in the morning at the time they normally wake up for work.

### 2.2 Sleep EEG data pre-processing

#### 2.2.1 Preprocessing and relative power calculation

We analyzed the relative spectral power of EEG in the sleep stages for nighttime and daytime sleep under dim and 30 lux environmental light conditions. The EEG signals were referenced to the contralateral mastoid. We performed 0.5–100 Hz band-pass filter using a Chebyshev digital filter. We removed ECG and EOG signal components using the EEGLAB toolbox from six-channel sleep EEG. If the power at a time point was higher than 5 standard deviations (SD), we considered it an artifact and removed it. When the proportion of artifacts in the given channel was more than 30% of the EEG data, we flagged it as a “bad” channel and replaced it with its opposite hemispherical channel data (for example, F3 could be replaced with F4, C3 with C4, and O1 with O2).

In our study, we utilized MNELAB, a Python-based processing toolbox, to analyze the spectral patterns of six-channel sleep EEG data (Gramfort et al., 2014) by applied a 3rd order Chebyshev bandpass filter to split the data into five frequency bands: slow (0.5–1 Hz), delta (1–4 Hz), theta (4–8 Hz), alpha (8–12 Hz), and sigma (12–15 Hz). The power in each frequency band was then computed. The sleep stages (N1–N3, REM) were manually scored according to the guidelines outlined by the American Academy of Sleep Medicine (AASM) standards (Berry et al., 2017). Subsequently, we calculated the spectral pattern of the sleep EEG data separately for each sleep stage. To enhance the spectral characteristics, we averaged the power values of the six-channel EEGs to obtain a single value. Finally, we determined the relative EEG power for each sleep stage by calculating the power ratio within a specific frequency band to the total power (0.5–15 Hz) of each participant. This approach, chosen over absolute power measurements, accounts for individual differences in factors such as skull thickness and scalp conductivity. Furthermore, relative power provides a more nuanced view of how specific frequency bands (e.g., delta or sigma waves) change in proportion to the overall EEG activity, amplifying alterations in specific types of brain activity.

#### 2.2.2 Sleep cycle separation

We segmented the sleep cycle, which initiates with NREM sleep and transitions into REM sleep as sleep becomes more profound (Carskadon and Rechtschaffen, 2011). Subsequently, we analyzed the changes in EEG spectral patterns corresponding to the progression of sleep. These comparisons were performed between nighttime and daytime sleep under dim light conditions, and between daytime sleep with dim light and nighttime sleep with 30 lux light exposure conditions. We developed a MATLAB toolbox for sleep cycle partitioning^1^ to visualize the sleep stages of the subjects and perform single clicks on the hypnogram to record the split points of different cycles.

The nighttime sleep group exhibited an average of 4.52 ± 0.79 cycles, ranging from 3 to 6 cycles. In the daytime sleep group exposed to 30 lux light, the average number of sleep cycles was 4.41 ± 0.77, also ranging from 3 to 6 cycles. Meanwhile, under the dim light setting, the daytime sleep group had an average of 4.57 ± 0.85 sleep cycles, also within a 3 to 6 cycles range. Because the minimum number of sleep cycles in the data was three and the number of cycles varied across individuals, to allow between-individual analysis, we extracted the first, second, and final sleep cycles from all individuals and analyzed them for EEG temporal pattern changes.

### 2.3 Statistical analysis

#### 2.3.1 Nighttime sleep with dim light vs. daytime sleep with dim light (<5 lux)

To explore how circadian misalignment affects sleep quality, we compared nighttime and daytime sleep under dim light conditions. First, we analyzed demographic and PSG variables using the Student’s independent sample *t*-test. We then conducted separate linear regression analyses to compare the relative power of electrophysiological activity for the entire night between nighttime and daytime sleep. This comparison was performed for five frequency bands and three NREM sleep stages. Additionally, we examined the relative EEG power between the two groups representing different sleep conditions for the first, second, and final sleep cycles using linear regression, again considering all five frequency bands and the three NREM sleep stages. Because the insomnia severity index (ISI) was significantly different between the two groups (Table 1; participants’ detailed characteristics are further reported in the Results section 3.1), we included it as a covariate in the EEG power comparison to correct for its possible effect. All statistical results were corrected for multiple comparisons by controlling for false discovery rate (FDR).

**Table 1:** Demographic and clinical characteristics of the participants.

Variables	Daytime sleep of night shift nurses (*n* = 31)	Nighttime sleep of day workers (*n* = 24)	*p*
Age, years	26.7 ± 3.10	26.9 ± 3.55	0.846
Female, %	100	100	1
BMI, kg/m^2^	20.4 ± 2.12	20.4 ± 1.94	0.989
Insomnia severity index (ISI)	15.8 ± 3.74	5.7 ± 2.97	< 0.001
Epworth sleepiness scale (ESS)	9.0 ± 2.87	8.1 ± 3.74	0.292

#### 2.3.2 Daytime sleep with dim light (<5 lux) vs. daytime sleep with 30 lux light condition

To examine the influence of light exposure on daytime sleep quality, we followed procedures similar to those described in section 2.3.1. However, paired t-tests were used instead of linear regression because the two conditions were tested within the same cohort. We compared the relative power of EEG for the entire night, and first, second, and final sleep cycles between dim light and 30 lux light conditions, considering the five frequency bands and three NREM sleep stages. As in the previous analysis, all statistical results were adjusted for multiple comparisons using the FDR control method.

## 3 Results

### 3.1 Participants’ characteristics

The average time of employment as shift nurses was 3.8 ± 2.74 years (range 1.2–14.3 years). The mean ISI score of the daytime sleep group was higher than that of the nighttime sleep group (15.8 ± 3.74 h vs. 5.7 ± 2.97 h, *p* < 0.001). The mean ESS scores did not differ significantly between the daytime and nighttime sleep groups (Table 1).

### 3.2 Comparison of PSG result between daytime sleep in night shift nurses with dim light and nighttime sleep in day workers

The daytime sleep group showed shorter sleep latency, higher percentage of wakefulness after sleep onset (WASO), lower sleep efficiency, reduced percentage of N2 sleep, and higher percentage of N3 sleep than the nighttime sleep group. The total arousal index was similar between the groups; however, the spontaneous arousal index was higher in the daytime sleep group. The subjectively estimated TST was longer in the daytime sleep group than in the nighttime sleep group. However, the TST for the nighttime sleep group, at 5.5 ± 1.41 h, was shorter than the time of 6.5 h suggested by previous studies (Patel et al., 2009; Åkerstedt et al., 2022). This discrepancy can be attributed to our nighttime sleep group being primarily hospital employees, as they were recruited from hospitals. The average TST for hospital workers has been reported to be 5.75 ± 0.74 h, which is consistent with our findings (Table 2; Choi et al., 2021).

**Table 2:** Polysomnography results during daytime sleep of shift workers and nighttime sleep of healthy adults.

Variables	Daytime sleep of night shift nurses (*n* = 31)			Nighttime sleep of day workers (*n* = 24)	
	Condition 1 (30 lux)	Condition 2 (< 5 lux)	*p* *1* vs. *2*	*p* *2* vs. Night	
Time in bed, h	5.6 ± 1.44	6.1 ± 1.47	**0.040**	5.7 ± 0.36	0.143
Total sleep time, h	5.1 ± 1.47	5.5 ± 1.41	0.149	5.4 ± 0.44	0.676
Sleep latency, min	2.3 ± 1.77	2.4 ± 3.45	0.869	4.6 ± 4.4	**0.038**
REM latency, min	57.5 ± 28.27	65.0 ± 31.56	0.236	83.2 ± 38.56	0.057
WASO, %	9.4 ± 7.33	9.7 ± 6.50	0.859	4.5 ± 2.72	**< 0.001**
Sleep efficiency, %	89.9 ± 7.33	89.6 ± 6.44	0.874	94.2 ± 3.27	**0.001**
N1 sleep, %	12.3 ± 6.35	11.1 ± 5.38	0.386	8.8 ± 4.37	0.079
N2 sleep, %	40.5 ± 9.72	40.7 ± 9.86	0.942	51.4 ± 7.11	**< 0.001**
N3 sleep, %	22.6 ± 10.80	25.0 ± 10.74	0.240	18.1 ± 7.63	**0.010**
REM sleep, %	24.6 ± 6.96	23.1 ± 5.67	0.333	21.7 ± 5.92	0.394
Total arousal index, /h	12.5 ± 4.92	12.9 ± 5.35	0.604	12.8 ± 5.50	0.931
Spont. Arousal index, /h	10.5 ± 5.02	11.1 ± 5.48	0.490	8.2 ± 4.25	**0.038**
Movement Arousal index, /h	0.3 ± 0.51	0.2 ± 0.49	0.220	1.1 ± 3.52	0.256
REM Arousal index, /h	10.4 ± 5.02	12.5 ± 4.93	**0.038**	15.0 ± 8.44	0.183
AHI, /h	1.2 ± 2.00	1.1 ± 2.15	0.619	2.2 ± 2.10	0.071
Estimated total sleep time, h	5.2 ± 1.37	5.9 ± 1.51	0.077	5.2 ± 1.65	**0.041**
Estimated sleep latency, min	12.9 ± 9.56	12.3 ± 9.48	0.752	15.3 ± 11.41	0.296

WASO, wakefulness after sleep onset; spont., spontaneous; AHI, apnea-hypopnea index; bold, significant.

### 3.3 PSG parameters during daytime sleep: dim light vs. 30 lux light condition

There were no significant differences in PSG parameters between the two light conditions except for time in bed and REM arousal index: Time in bed and REM arousal index in the dim light condition was longer than that in the 30 lux light exposure condition (Table 2).

### 3.4 Relative power differences between dim light nighttime sleep and dim light daytime sleep

**Figure 1:**
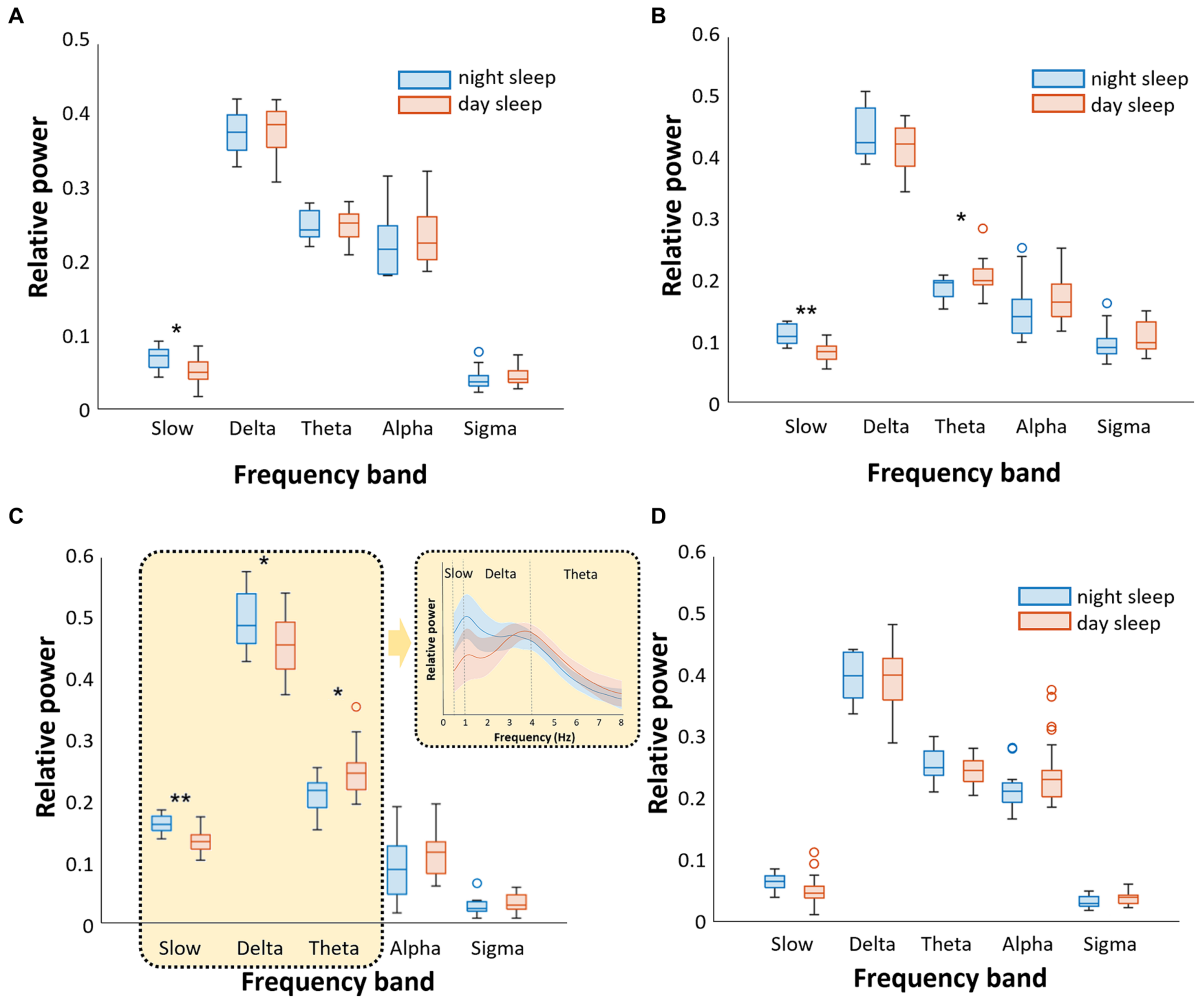
Comparison of the relative power of sleep EEG between nighttime sleep and daytime sleep [both under dim light conditions (<5 lux)]. We analyzed separately five frequency bands (slow, delta, theta, alpha, and sigma) and four sleep stages **(A)**. In N1 sleep, slow wave was higher during nighttime sleep **(B)**. In N2 sleep, slow wave was higher and theta wave was lower during nighttime sleep **(C)**. In N3 sleep, slow and delta waves were higher, while theta wave was lower during nighttime sleep (D). There were no significant differences in REM sleep (*p < 0.05, **p < 0.001).

The relative power characteristics of EEG in the N1-N3 and REM sleep stages between nighttime and daytime sleep under dim light conditions are shown in Figure 1. In the N1 sleep stage, the relative power of the slow wave was significantly lower during daytime sleep with dim light than during nighttime sleep (*p* < 0.05). Other frequency bands did not differ between the two groups (Figure 1A). In the N2 sleep stage, the relative power of the slow wave was significantly lower, and the relative power of the theta band was higher during daytime than during nighttime sleep (*p* < 0.05, Figure 1B). In the N3 sleep stage, the relative powers of the slow and delta waves were significantly lower during daytime sleep than during nighttime sleep (slow: *p* < 0.001; delta: *p* < 0.05), and the relative power of the theta waves was significantly higher during daytime than during nighttime sleep (*p* < 0.05). In-depth visualization of the relative power in the narrow band from 0 to 8 Hz (the bands of slow, delta, and theta waves) showed that the nighttime sleep group exhibited a peak relative power at approximately 1 Hz between the slow and delta bands, whereas the daytime sleep group showed a peak at approximately 4 Hz between the delta and theta bands (Figure 1C). This suggests that the poorer N3 sleep for daytime sleepers is due to a lack of slow-wave activities and more promoted theta wave activities compared to nighttime sleepers.

During REM sleep, there were no differences in the relative powers of all frequency bands between nighttime and daytime sleep (Figure 1D).

### 3.5 Relative power differences between dim light and 30 lux light exposure during daytime sleep

**Figure 2:**
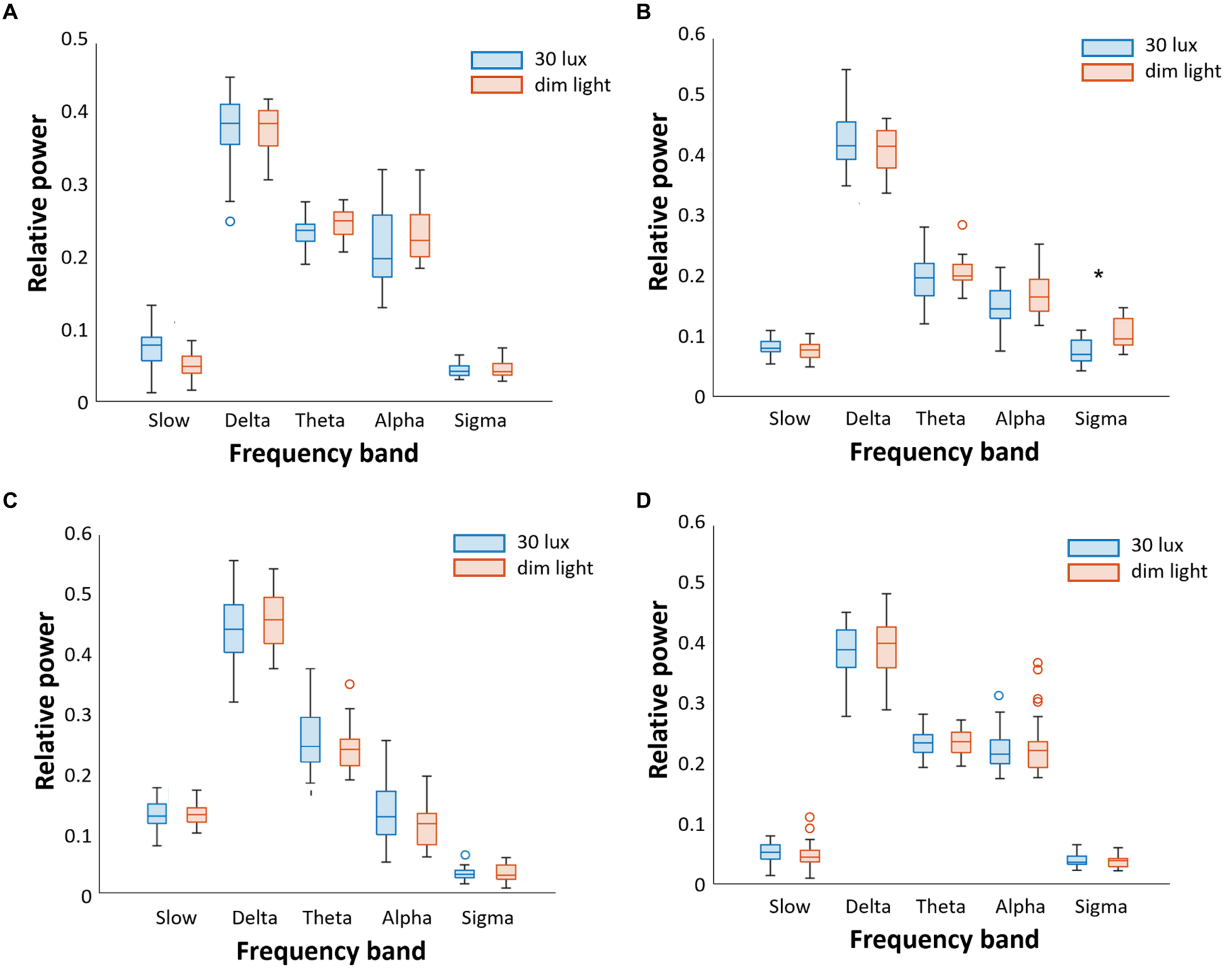
Comparison of the relative power of sleep EEG between 30 lux and dim (<5 lux) light conditions during daytime sleep. We separately analyzed five frequency bands (slow, delta, theta, alpha, and sigma) and four sleep stages **(A)**. In N1 sleep, there were no significant differences between 30 lux and dim light conditions **(B)**. In N2 sleep, sigma wave activity was lower in the 30 lux light condition **(C)**. In N3 sleep, there were no significant differences **(D)**. There were no significant differences in REM sleep either (*p < 0.05).

The relative power of EEG differences in the N1-N3 and REM sleep stages under different ambient light conditions is shown in Figure 2. In the N2 sleep stage, the relative power of the sigma wave, which is the main band frequency of sleep spindles, was significantly higher under dim light than under 30 lux light condition (*p* < 0.05) (Figure 2B). Other frequency band waves were not significantly different between the dim light and 30 lux light conditions. There were no differences in the relative power of all frequency bands between the dim light and 30 lux light conditions in the N1, N3, and REM sleep stages (Figures 2A,C,D).

When the time group was used as a covariate in the linear regression analysis, there was no change in the results. Absolute power was measured across all EEG frequency bands, and comparisons between groups were conducted. These results are presented in Supplementary Table S1.

### 3.6 Temporal patterns of slow-wave power varying across sleep cycles: comparison between different sleep groups and different light conditions

**Figure 3:**
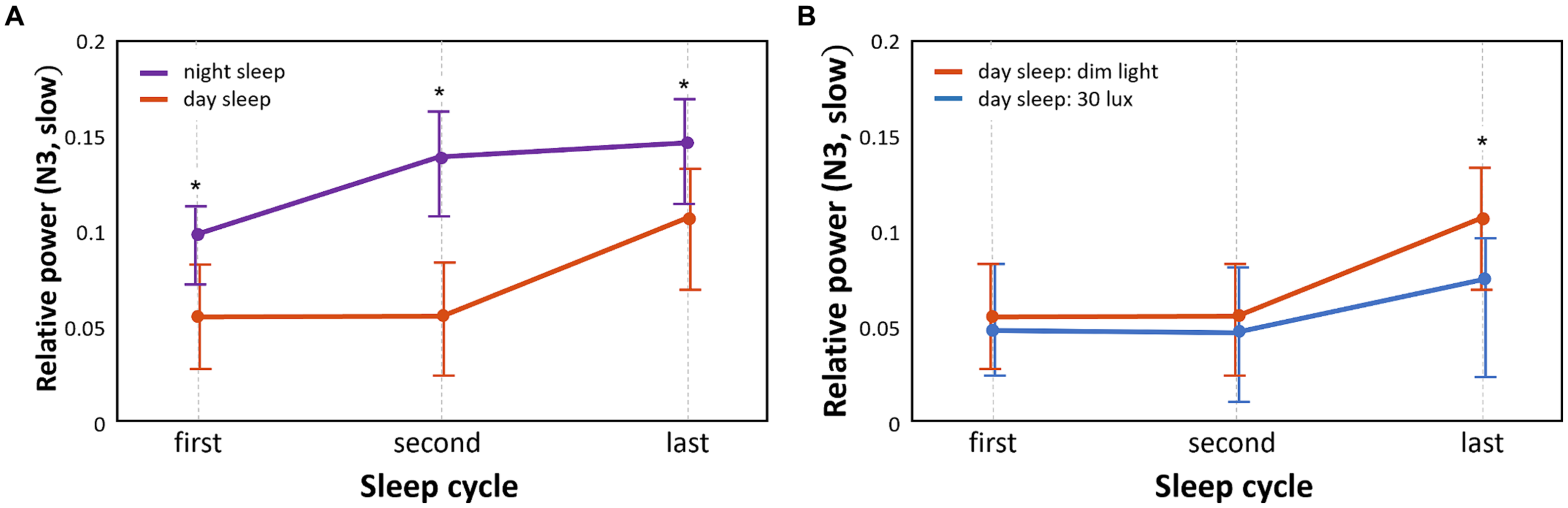
Comparison of temporal patterns of N3 slow wave power between different sleep groups and between different light conditions. We separately analyzed the N3 slow wave in each of the three sleep cycles (first, second, and last) (*p < 0.05) **(A)**. Relative power differences between nighttime sleep and daytime sleep under the same dim light conditions **(B)**. Relative power differences between 30 lux and dim light conditions during daytime sleep.

The pattern of relative EEG power of slow waves that varied with the sleep cycle in the different groups is shown in Figure 3. The relative EEG power of N3 sleep during daytime sleep with dim light was significantly lower than that during nighttime sleep with dim light in the first, second, and last cycles (*p* < 0.05; Figure 3A). Frequency bands in sleep stages other than N3 did not show significant differences between daytime and nighttime sleep with dim light conditions.

The relative power of slow waves in daytime sleep with dim light compared to daytime sleep with 30 lux light was significantly higher (*p* < 0.05) in N3 sleep of the last sleep cycle only (Figure 3B). Frequency bands in sleep stages other than N3 did not show significant differences between the dim and 30 lux light conditions during daytime sleep.

## 4 Discussion

In this study, the analysis of sleep EEG power showed a lower relative power of slow or delta waves in all NREM sleep stages (N1: slow, N2, and N3: slow and delta) during daytime sleep in shift workers compared to nighttime sleep in day workers, whereas a higher power in the theta wave in the N2 and N3 stages was observed during daytime sleep compared to nighttime sleep. We found that during daytime sleep, a lower sigma wave power in N2 was observed under light exposure conditions compared to no light exposure. Moreover, daytime sleepers had lower slow wave power in the N3 stage in all three cycles (first, second, and last) than nighttime sleepers. During daytime sleep, a lower slow wave power in the N3 stage of the last cycle was observed under light exposure than under no light exposure.

### 4.1 Differences in PSG parameters and relative power of EEG between daytime sleep with dim light and nighttime sleep with dim light

Rotating shifts are common among nurses to ensure the continuity of care. One of the most harmful effects of night-shift work is the deterioration of sleep patterns caused by circadian rhythm disruption and increased sleep homeostatic pressure (Wright et al., 2013; Alfonsi et al., 2021). Despite the increase in sleep pressure as a result of night shifts, it has been reported that the effect of light exposure during night work can lead to poor-quality daytime sleep (Geiger-Brown et al., 2012; Wright et al., 2013; Kaliyaperumal et al., 2017; Sletten et al., 2017; Sunde et al., 2020). Persistent poor sleep may lead to increased fatigue, which can negatively impact patient and staff safety. Therefore, it is essential to create a low-risk environment for sleep, possibly through the use of eye masks, earplugs, and light-blocking curtains or shades (Wright et al., 2013).

Our comparison of other PSG parameters between daytime and nighttime sleep under the same dim light conditions showed shorter sleep latency, higher percentage of WASO, lower sleep efficiency, reduced percentage of N2 sleep, and higher percentage of N3 sleep during daytime sleep than during nighttime sleep. This finding is reasonable because when sleep deprivation occurs owing to night work, the homeostatic drive for sleep increases (Boivin et al., 2022), which may further decrease sleep latency and increase the percentage of N3 sleep. Similar results were also found in a simulation study by Pedersen et al. (2022). On the contrary, our findings of higher percentage of WASO and lower sleep efficiency during daytime sleep suggest that sleep disturbances are common in shift workers, likely due to circadian rhythm misalignment, resulting in low melatonin levels and high cortisol levels during daytime sleep (Boivin et al., 2022).

When comparing the relative spectral power of sleep EEG between daytime and nighttime sleep, the daytime sleep group had a significantly lower power of slow waves in the entire NREM sleep period (N1, N2, and N3) and a lower power of delta waves in N3 sleep. This is supported by previous studies, which showed that shift workers’ daytime sleep inhibits slow-wave activity compared with normal nighttime sleep (Grønli et al., 2017).

The daytime sleep group also presented a significantly higher theta wave power during N2 and N3 sleep. In contrast, the percentage of N3 sleep during daytime sleep was greater than that during nighttime sleep. Despite this increased percentage of longer N3 sleep during daytime sleep, it is noteworthy that spectral power analysis indicated that the quality of N3 sleep in daytime sleepers may be compromised due to the lower power in slow and delta waves, as demonstrated in Figure 1C. The impact of this altered power pattern of N3 sleep during daytime sleep on night-shift work performance remains to be clarified.

### 4.2 Differences in the relative power of EEG between dim light and 30 lux light conditions during daytime sleep

There were no significant differences in PSG parameters between dim light (<5 lux) and overhead room light (30 lux) conditions during daytime sleep, except for the mean time in bed and the REM arousal index. The first night shift in a sequence often leads to prolonged wakefulness (Ganesan et al., 2019). By the end of the first night, the continuous time awake often reaches 24 h or longer and coincides with the circadian nadir for alertness (Ganesan et al., 2019). For this reason, it is possible that light stimuli have only subtle effects on PSG parameters. Thus, these subtle changes in PSG are reasonably different from the strong effects of light exposure on normal nighttime sleep in day workers (Cho et al., 2013).

Thus, our in-depth analysis explored the possibility of alterations in the spectral power of sleep EEG waves for various sleep stages and cycles in relation to light exposure during daytime sleep Indeed, we identified decreased power of sleep spindles (represented by the sigma wave) in N2 sleep for night-shift workers with light exposure during daytime sleep compared to when they slept without light exposure during daytime. Sleep spindles serve as a transitional phase between the light sleep of stage 1 and the deep sleep stages 3, known as slow-wave sleep. Their contribution to the cyclical pattern of sleep stages is crucial for upholding a stable sleep architecture (De Gennaro and Ferrara, 2003; Andrillon et al., 2011; Purcell et al., 2017). Sleep spindles result from an interaction between the thalamus and the cortex. This interaction is crucial for the synchronization of neuronal activity, which is important for the transition between sleep stages and the maintenance of a stable sleep state (Steriade, 2006; Astori et al., 2013; Lüthi, 2014). Any disruptions to this interaction can result in fragmented sleep and diminished sleep quality, which suggests that light exposure may disturb sleep stability. Furthermore, sleep spindle activity is highly associated with memory consolidation (Bergmann TOMolle et al., 2012; Leminen et al., 2017). Future studies are required to clarify whether light-exposed daytime sleep leads to impaired memory consolidation.

We did not observe differences in slow waves during N3 sleep between the dim light and 30 lux light conditions. This is likely explained by our finding that slow waves significantly decrease during daytime sleep with dim light. Therefore, daytime sleep strongly promotes poor deep sleep, and subsequently, no further decrease in slow waves could be exerted by light exposure.

However, our in-depth analysis of different sleep cycles showed that light exposure during daytime sleep significantly decreased the relative power of slow waves in the last cycle, but not in the early cycles (i.e., first and second cycles).

### 4.3 Limitations

The current study had some limitations. First, the sample size was relatively small, with 31 night shift nurses and 24 daytime workers. Secondly, there was no significant difference in TST between daytime sleepers (5.4 ± 0.44 h) and nighttime sleepers (5.5 ± 1.41 h). In contrast, previous research has reported an average nighttime TST of approximately 6.5 h (Patel et al., 2009; Åkerstedt et al., 2022), which is longer than that observed for nighttime sleepers. The shorter TST in our results for nighttime sleepers might be attributed to the fact that they were recruited from hospitals and were predominantly hospital employees. Their average nighttime TST was reported to be 5.75 ± 0.74 h, aligning with our observations (Choi et al., 2021). Thirdly, the electrophysiological activity changes during daytime sleep may vary depending on the chronotype. Previous studies on nighttime sleep have shown that morning sleepers had fewer slow waves and more sigma wave activities in NREM compared to evening sleepers (Mongrain et al., 2005, 2006). In the current study, the relationship between chronotype and night shift work, circadian rhythm shift, or exposure to ambient light was not investigated; therefore, future studies may need to take this into account. Fourth, our study was based solely on young female participants working in a hospital environment, leading to a potential bias in our analysis. Furthermore, it is noteworthy that these nurses were not fixed night-shift workers but were in a 12-h rotation system. The generalizability of this study may be improved by including male participants, other age groups, fixed night-shift workers, and other occupations. Fifth, the effects of night-shift work (where nighttime sleep deprivation occurs and light exposure occurs during nighttime work) and circadian rhythm shifts could not be separately analyzed, although both can cause EEG power changes during daytime sleep. Finally, although we compared daytime sleep with and without light exposure, our analysis did not compare the effects of light exposure on daytime or nighttime sleep. This may not completely distinguish the effects of light exposure from the effects of circadian rhythm shift or night-shift work.

## 5 Conclusion

Our research clearly indicates that night shift work and the resulting disruption of the body’s natural sleep–wake cycle have a significant impact on sleep quality. Specifically, we found that these factors lead to a decrease in slow wave and delta wave activities during NREM sleep. Additionally, we discovered that exposure to light during daytime sleep further reduced N2 sleep spindle activity and decreased N3 wave activity, particularly during the final sleep cycle. These findings highlight the negative effects of shift work and daytime light exposure on the sleep quality of individuals working night shifts.

## Data availability statement

The datasets for this article are not publicly available due to concerns regarding participant/patient anonymity. Requests to access the datasets should be directed to the corresponding author.

## Ethics statement

The studies involving humans were approved by Institutional Review Board of Samsung Medical Center (IRB No:2018-05-120 for daytime sleep in shift workers and 2018-10-037 for nighttime sleep in healthy adults). The studies were conducted in accordance with the local legislation and institutional requirements. The participants provided their written informed consent to participate in this study.

## Author contributions

SY: Formal analysis, Methodology, Validation, Writing – original draft. SC: Conceptualization, Data curation, Validation, Writing – original draft. CZ: Formal analysis, Methodology, Software, Writing – original draft. EJ: Funding acquisition, Project administration, Supervision, Writing – review & editing. HK: Conceptualization, Supervision, Writing – review & editing.

## Footnotes

^1^GitHub link: https://github.com/ZCONG2025/Sleep-Cycle-Separation/tree/main
