# Supplementary material for: Are there effects of light exposure on daytime sleep for rotating shift nurses after night shift?: an EEG power analysis
Source: Front Neurosci. 2024 Mar 27;18:1306070. doi: 10.3389/fnins.2024.1306070 (PMC11004303; doi:10.3389/fnins.2024.1306070)
Supplement: Supplementary file 1 [file Data_Sheet_1.docx]

Supplementary Figure 1. Flow chart of shift worker study design


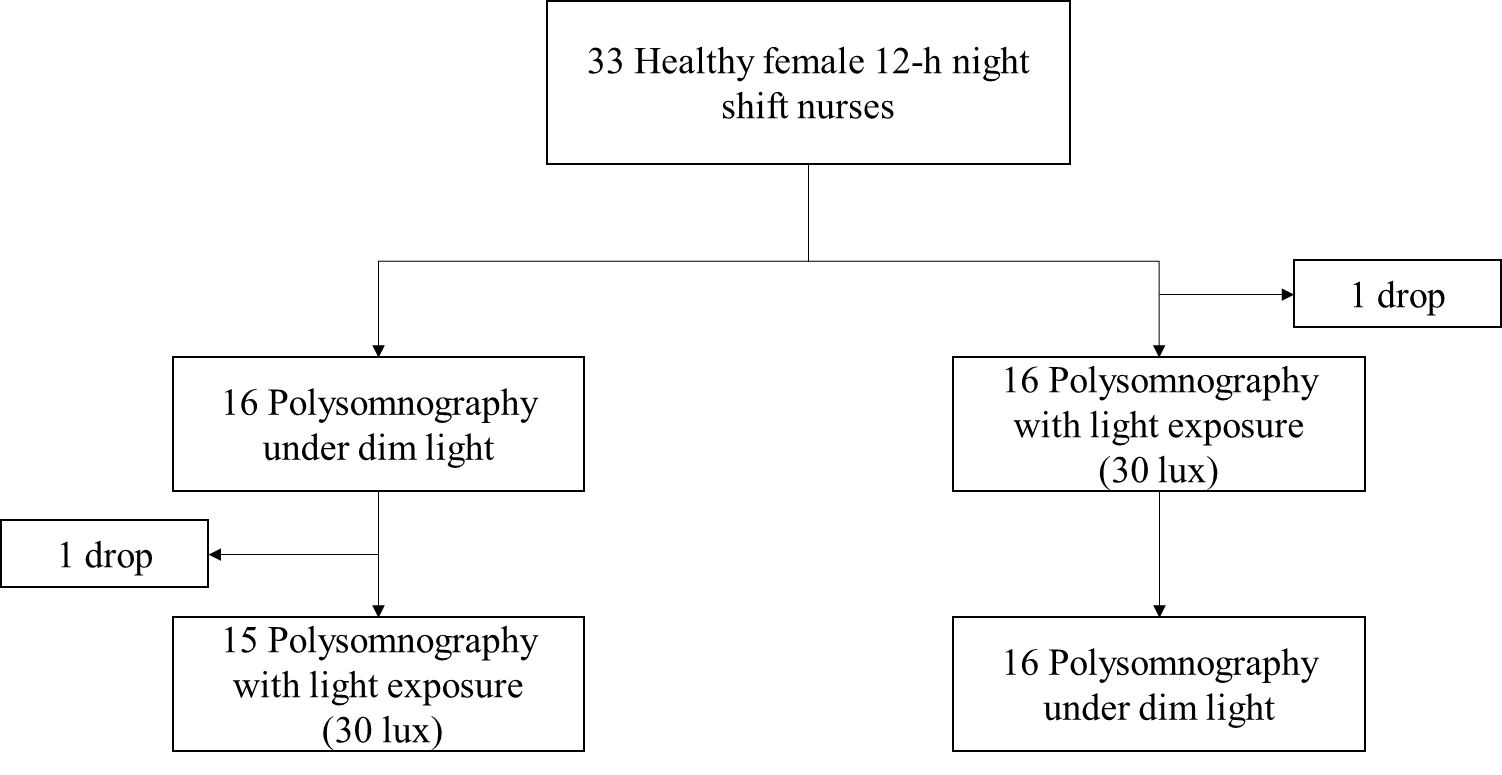


Supplementary Table 1. Comparison of absolute power across all sleep stages and EEG bands for night sleep with dim light condition vs. day sleep with dim light condition, and day sleep with dim light condition vs. day sleep with 30 lux light condition

|  | Night sleep | | | | | Day sleep | | | | | | | | | | *P*-value | | | | | | | | | | |
| --- | --- | --- | --- | --- | --- | --- | --- | --- | --- | --- | --- | --- | --- | --- | --- | --- | --- | --- | --- | --- | --- | --- | --- | --- | --- | --- |
|  | Dim^1^ | | | | | Dim^2^ | | | | | 30lux^3^ | | | | | 1 vs. 2 | | | | | 2 vs. 3 | | | | | |
| Sleep stage | Slow | Delta | Theta | Alpha | Sigma | Slow | Delta | Theta | Alpha | Sigma | Slow | Delta | Theta | Alpha | Sigma | Slow | Delta | Theta | Alpha | Sigma | Slow | Delta | Theta | Alpha | Sigma |  |
| N1 | 7.0E-10 | 2.4E-09 | 9.3E-10 | 9.1E-10 | 2.9E-10 | 5.8E-10 | 2.3E-09 | 1.0E-09 | 1.0E-09 | 2.5E-10 | 5.8E-10 | 2.4E-09 | 1.1E-09 | 9.6E-10 | 2.5E-10 | 0.009 | 0.036 | 0.275 | 0.321 | 0.538 | 0.80 | 0.81 | 0.85 | 0.86 | 0.85 |  |
| N2 | 5.1E-09 | 1.8E-08 | 7.0E-09 | 6.1E-09 | 3.2E-09 | 2.3E-09 | 1.0E-08 | 5.2E-09 | 4.3E-09 | 2.0E-09 | 2.2E-09 | 9.6E-09 | 4.6E-09 | 4.0E-09 | 1.8E-09 | <0.0001 | 0.003 | 0.214 | 0.032 | 0.024 | 0.77 | 0.50 | 0.40 | 0.56 | 0.65 |  |
| N3 | 8.3E-10 | 3.6E-09 | 1.9E-09 | 1.5E-09 | 4.1E-10 | 5.4E-10 | 3.5E-09 | 2.7E-09 | 1.8E-09 | 4.8E-10 | 4.8E-10 | 3.2E-09 | 2.3E-09 | 1.6E-09 | 4.2E-10 | <0.0001 | <0.0001 | <0.0001 | 0.009 | 0.004 | 0.45 | 0.48 | 0.34 | 0.27 | 0.42 |  |
| REM | 2.2E-09 | 8.1E-09 | 3.3E-09 | 2.8E-09 | 1.3E-09 | 1.1E-09 | 5.4E-09 | 3.0E-09 | 2.4E-09 | 9.0E-10 | 1.1E-09 | 5.1E-09 | 2.7E-09 | 2.2E-09 | 8.3E-10 | 0.011 | 0.390 | 0.513 | 0.364 | 0.646 | 0.67 | 0.60 | 0.53 | 0.56 | 0.64 |  |
